# Determine neighboring region spatial effect on dengue cases using ensemble ARIMA models

**DOI:** 10.1038/s41598-021-84176-y

**Published:** 2021-03-12

**Authors:** Loshini Thiruchelvam, Sarat Chandra Dass, Vijanth Sagayan Asirvadam, Hanita Daud, Balvinder Singh Gill

**Affiliations:** 1 Insititute of Autonomous Systems, Universiti Teknologi PETRONAS, Seri Iskandar, Perak Malaysia; 2 School of Mathematical and Computer Sciences, Heriot–Watt University Malaysia, Putrajaya, Malaysia; 3 Department of Electric and Electronic Engineering, Universiti Teknologi PETRONAS, Seri Iskandar, Perak Malaysia; 4 Department of Fundamental and Applied Sciences, Universiti Teknologi PETRONAS, Seri Iskandar, Perak Malaysia; 5 Institute for Medical Research (IMR), Ministry of Health, Kuala Lumpur, Malaysia

**Keywords:** Diseases, Infectious diseases

## Abstract

The state of Selangor, in Malaysia consist of urban and peri-urban centres with good transportation system, and suitable temperature levels with high precipitations and humidity which make the state ideal for high number of dengue cases, annually. This study investigates if districts within the Selangor state do influence each other in determining pattern of dengue cases. Study compares two different models; the Autoregressive Integrated Moving Average (ARIMA) and Ensemble ARIMA models, using the *Akaike* Information Criterion (AIC) and Bayesian Information Criterion (BIC) measurement to gauge their performance tools. ARIMA model is developed using the epidemiological data of dengue cases, whereas ensemble ARIMA incorporates the neighbouring regions’ dengue models as the exogenous variable (X), into traditional ARIMA model. Ensemble ARIMA models have better model fit compared to the basic ARIMA models by incorporating neighbuoring effects of seven districts which made of state of Selangor. The AIC and BIC values of ensemble ARIMA models to be smaller compared to traditional ARIMA counterpart models. Thus, study concludes that pattern of dengue cases for a district is subject to spatial effects of its neighbouring districts and number of dengue cases in the surrounding areas.

## Introduction

Dengue is a vector-borne disease, transmitted by two types of mosquito vectors; the *Aedes Aegypti* and *Aedes Albopictus,* where the life-cycle of the vector and transmission of the disease are closely related to climate variables^1^. Dengue is endemic in tropical and subtropical regions worldwide, and this includes Malaysia, specifically the state of Selangor^2^. Of the total number of 120,836, 101,357 and 83,849 dengue cases that occurred in Malaysia, during the years of 2015, 2016, and 2017 respectively, 52.30%, 50.96% and 54.00% of these cases occurred in the state of Selangor^3^. The state level health authorities would alert all the districts in the abovementioned states (namely, Petaling, Klang, Hulu Selangor, Hulu Langat, Kuala Selangor, Gombak, Sepang, Kuala Langat and Sabak Bernam) if there is/are always hotspot(s) or many confirmed dengue cases being identified within these localities^4^.

These *Aedes* mosquito vector populations are difficult to control and to eliminate as their eggs can mature and hatch even after a prolonged drought^1^. However, control against the mosquito vectors is known to be the best measure for curbing the rise of dengue cases at present. Vaccination for this disease is still under study as the efficacy of the vaccine has yet to yield reliable outcomes. One such example is dengue vaccine named *Dengvaxia* which has been developed by Sanofi Pasteur Ltd, but further investigations has found this vaccine was only effective for those who have been infected previously with dengue, however may cause a more severe disease and hospitalization to those who are sero-negative (not being infected prior)^5^.

In view of these developments, forecasting increases in dengue cases is still relevant and important for health authorities in Malaysia. Dengue prediction models are an important complementary tools to determine when an increase in dengue cases will occur and thereby to deploy methods of controlling the vector population early.

In looking into possible factors to be included in dengue prediction models, a previous study^6^ emphasized six factors contributing to dengue increase: serotype shift, climate change, human behavior, poor environmental sanitation, *mobility of population* and the ineffectiveness of the vector control activities. Therefore, these factors mentioned should be considered while building the dengue forecasting models as studies have suggested that the circulating serotype contributes to the rising trend of dengue cases^7–9^. However determining the circulating serotype requires laboratory testing which is time consuming compared to the climate factors. Moreover weather based dengue early warning surveillance systems are considered as simple, low cost, and more efficient systems^10^. Several dengue affected countries also have studied dengue-climate relationships and incorporated climate variables into their dengue forecasting models^9^.

Besides the climate, several studies have also discussed the spatial aspects of dengue patterns in developing effective prediction models. These studies discussed the global dengue prediction models^11,12^ and also spread across states^13^, districts^14,15^ and at localized levels^16–18^. Studies have shown that dengue prediction models are best to be localized to distribution of the mosquito vector populations that varies with biological and environmental factors^15^. Several different scales of localization have been used by previous studies, for example one study investigated dengue patterns with a range of only 100 m within houses of children suspected to have fever^18^, which represents the distance that female *Aedes Aegypti* mosquitoes could fly up to. Further expanding this distance, previous studies have clustered dengue incidences as hotspots within a range of 100 m–1 km^19,20^. Here, the direct chain of dengue transmission, flight range of the mosquito, human movement within localities and the similar serotype-specified immunity acquired by the respective residents were considered as contributing factors.

Movement of dengue-infected individuals would result in increasing the size of the outbreak clusters where many studies have instead considered localization at district levels instead of localities. A study carried out in Southern Vietnam found that high and low numbers of dengue cases occurred at time points close to each other for districts located within 100km^2^ radius, with villages located at a shorter distance of 52km^2^ radius of each other having more temporal coorelations^15^. In addition, a study in Taiwan suggested that temporal coorelations in neighbouring areas can be used to determine the risk of a dengue outbreak occuring in future^21^. The authors reported that the risk of an outbreak occuring in a area corresponds to the the probability of its neighbouring areas having a higher number of dengue cases. This possibility is measured as indices over time (temporal indices) and a total of three indices are used, consisting of probability for dengue occurrences, duration of an epidemic and the intensity of an endemic. Therefore, with these examples, it can be suggested that dengue cases are subject to spatial effect too, that is, the pattern of dengue cases in an area can be influenced by the cases in neighbouring areas. Considering the importance of neighbouring regions, this study aims to investigate spatial effects for the selected study area. For this purpose, this study applies two Box-Jenkins based model structures, the Autoregressive Integrated Moving Average (ARIMA) and its extension, Autoregressive Integrated Moving Average with Exogenous variable (ARIMAX) models (described as Ensemble ARIMA for this study) for predicting dengue cases^22,23^.

Thus, specifically this study consists of three research objectives. First, is to build a single ARIMA dengue prediction model. The next step is to build an ensemble ARIMA model that incorporates the spatial effects of neighboring regions which represents an ARIMAX model structure with the ARIMA representing own regions’ dengue estimation model, and the external variables (X) from the neighboring regions’ dengue estimation models. Finally, the third objective is to compare and select the optimum dengue prediction models for each study region for single and ensemble model using AIC (*Akaike* Information Criterion) and BIC (Bayes Information Criterion) model selection tools.

## Materials methods

### Study region

**Figure 1:**
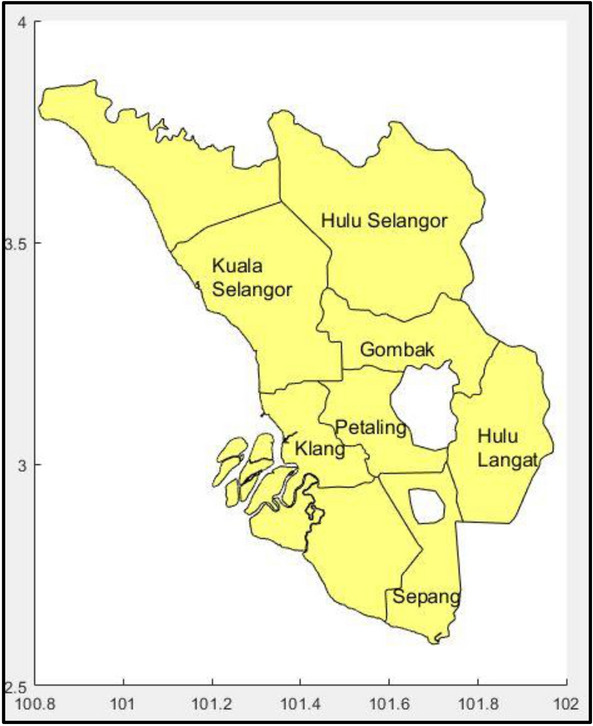
Locations of study districts in Selangor, Malaysia.

The study region consists of seven districts in the state of Selangor, Malaysia. These districts are, Hulu Selangor, Petaling, Klang, Kuala Selangor, Hulu Langat, Gombak and Sepang. Data on dengue cases were obtained from the Ministry of Health (MoH), Malaysia. The data used is on a weekly basis and was collected from the years 2009–2013, consisting of 260 weeks. Since the dengue trajectories are non-stationary, first order differencing with d = 1 is applied to convert dataset to stationary time series. The ARIMA and ARIMAX methodologies were then applied to the resulting differenced and stationary time series. It is found that d = 1 was sufficient to achieve stationarity and thus, higher order differencing was not needed. Figure 1 shows the study districts whereas the neighbouring regions for each study district which is needed to develop the ensemble models as in Eq. (2), is described in Table 1. These neighboring districts are aligned with a previous study emphasizing dengue cases within regions around 52km^2^ in average, are more likely to be correlated and their patterns may influence one area to another^15^. Thus, this study considered districts within 52km^2^ as the neighbouring districts and the distance on road are computed.

**Table 1:** Districts and their respective neighbouring(s) districts.

No	District	Nearest neighbour/s and distance (from respective district by road)
1	Hulu Selangor	Gombak (43km^2^)
2	Petaling,	Kuala Selangor (51km^2^), Gombak (33km^2^), Hulu Langat (52km^2^), Klang (30km^2^)
3	Klang	Kuala Selangor (49km^2^), Petaling (30km^2^)
4	Kuala Selangor	Gombak (55km^2^), Petaling (52km^2^), Klang (49km^2^)
5	Hulu Langat	Gombak (38km^2^), Petaling (52km^2^), Sepang (49km^2^)
6	Gombak	Hulu Selangor (43km^2^), Kuala Selangor (55km^2^), Hulu Langat (38km^2^), Petaling (33km^2^)
7	Sepang	Hulu Langat (49km^2^)

## ARIMA and ensemble ARIMA models for dengue prediction

In order to determine best dengue prediction model, a comparison study is carried out between models with (i) dengue cases of own region only and (ii) dengue cases of own region together with dengue models of neighbouring regions. These models are represented as the ARIMA and ensemble ARIMA (which can be viewed as ARIMAX extension) models, respectively, and developed using the Box-Jenkins (BJ) approach consisting of three stages: Model Identification, Parameter Estimation and Residual Diagnostics.

**Figure 2:**
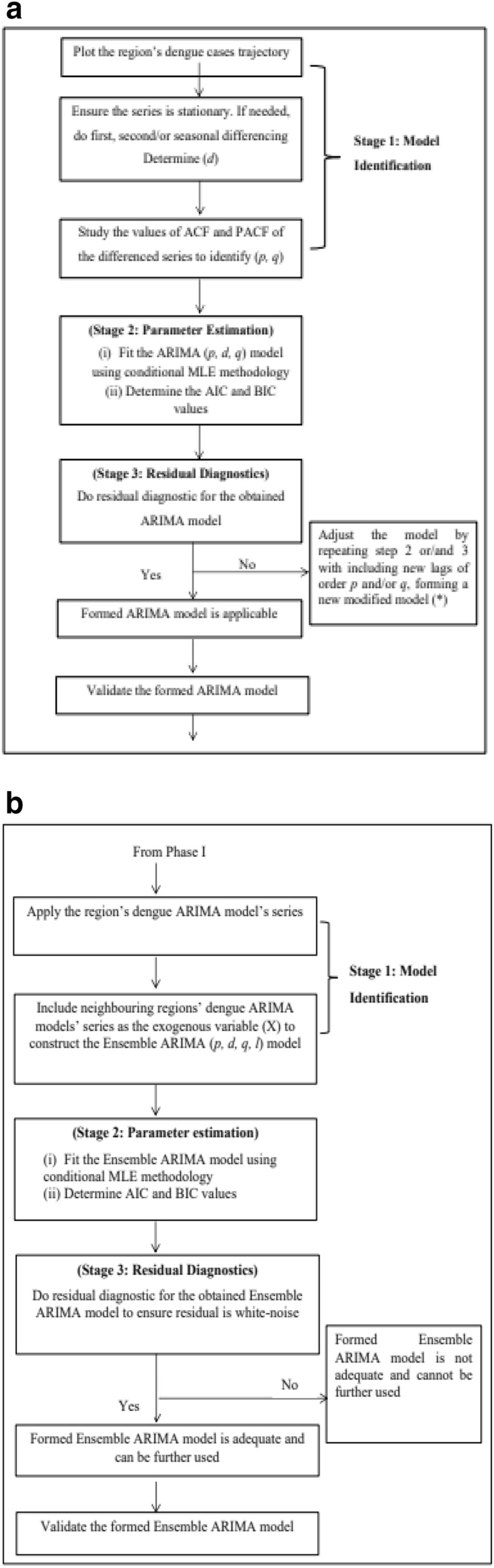
(**a**) Flow-chart of Box–Jenkins approach for ARIMA model development. (**b**) Flow-chart of Box–Jenkins approach for ensemble ARIMA model development.

Both ARIMA and Ensemble ARIMA models can be represented as Eqs. (1) and (2) respectively.

1 $${Y}_{t}^{{\prime}}={\phi }_{1}{Y}_{t-1}^{{\prime}}+{\phi }_{2}{Y}_{t-2}^{{\prime}}\dots +{\phi }_{p}{Y}_{t-p}^{{\prime}}+{\varepsilon }_{t}-{\theta }_{1}{\varepsilon }_{t-1}-{\theta }_{2}{\varepsilon }_{t-2}-\dots -{\theta }_{q}{\varepsilon }_{t-q}$$

2 $${{Y}_{t}^{*}}^{{\prime}}=\sum_{l=1}^{{n}_{b}}{\beta }_{i}^{\left(l\right)}{X}_{i}^{{{\prime}}\left(l\right)}+{\phi }_{1}{\widehat{Y}}_{t-1}^{{\prime}}+{\phi }_{2}{\widehat{Y}}_{t-2}^{{\prime}}+\dots +{\phi }_{p}{\widehat{Y}}_{t-p}^{{\prime}}+\widehat{{\varepsilon }_{t}}-{\theta }_{1}{\widehat{\varepsilon }}_{t-1}-{\theta }_{2}{\widehat{\varepsilon }}_{t-2}-\dots {-\theta }_{q}{\widehat{\varepsilon }}_{t-q}$$

where: ${\widehat{Y}}_{t-1}^{{\prime}}$ is the observation at time $(t-1)$ obtained from its own region’s dengue model, ${X}_{i}^{{{\prime}}(l)}$ are the observation obtained from the *i*-indexed dengue models of $\left(l\right)$ number of neighbouring regions’ dengue models and $\widehat{{\varepsilon }_{t}}$ is the error value at time $t$ obtained from its own region dengue model. The vectors $\underset{\_}{\phi }=\left({\phi }_{1}, {\phi }_{2},\dots ,{\phi }_{p}\right)$, $\underset{\_}{\theta }=\left({\theta }_{1},{\theta }_{2},\dots ,{\theta }_{q}\right)$ and $\underset{\_}{\beta }=\left({\beta }_{1}^{l}, {\beta }_{2}^{l}, \dots ,{\beta }_{i}^{l}\right)$ are the total $\left(p+q+l\right)$ unknown parameters to be estimated with each ${\phi }_{i}\in \mathfrak{R}$ in Eqs. (1) and (2) representing the correlation for previous dengue cases, ${\theta }_{i}\in \mathfrak{R}$ in Eqs. (1) and (2) representing the correlation for moving average of previous error terms to be included and finally ${\beta }_{i}\in \mathfrak{R}$ representing the correlation coefficient for the neighbouring regions’ dengue models considering each $\left(l\right)$. The parameters are estimated using the Maximum Likelihood (ML) approach, and residual diagnostics are carried out on the obtained models to ensure the errors are white-noise. The developments of ARIMA and Ensemble ARIMA models are illustrated in Fig. 2a,b, respectively.

### Akaike Information Criterion (AIC) and Bayes Information Criterion (BIC)

As two models are obtained for each study region, the optimum model needs to be identified, and therefore, the AIC and BIC values are used as the model selection tools. The best model would be the one with lowest AIC and BIC values. Since AIC safeguards against underfitting and BIC against overfitting, by considering both these AIC and BIC values, the selected model will have good prediction capability and will be parsimonious^24^.

The AIC and BIC values^25^ are defined as in Eqs. (3) and (4) respectively:

3 $$\begin{aligned} AIC & = - 2\left( {\log L} \right) + A_{n} *k \\ & = - 2\left( {\log L} \right) + 2*k \\ \end{aligned}$$

4 $$\begin{aligned} BIC & = - 2\left( {\log L} \right) + B_{n} *k \\ & = - 2\left( {\log L} \right) + \log\left( N \right)*k \\ \end{aligned}$$

where $\mathrm{log} \,L$ is the model likelihood, $k$ is the number of model parameters and $N$ is the number of observations in the time series data. The notation $k$ is used as follows: $k={k}_{0}+1$, where ${k}_{0}$ is the number of significant non-zero $\underset{\_}{\upphi }$ and $\underset{\_}{\uptheta }$ coefficients in the ARIMA ${\left(\mathrm{p},\mathrm{d},\mathrm{q}\right)}^{*}$ model (see Fig. 2a), the $\underset{\_}{\upphi }$ coefficients are represented by at most $p$ non-zero values and the $\underset{\_}{\uptheta }$ coefficients are represented by at most $q$ non-zero values. The extra $\left("+1"\right)$ corresponds to the variance parameter, ${\upsigma }^{2}$. For the Ensemble ARIMA $\left(p,d,q,l\right)$ model, the value of k is $\mathrm{k}={\mathrm{k}}_{0}+\mathrm{m}+1$ since there are $m$ number of $\beta$-coefficients of neighbouring regions’ dengue models and one variance parameter,${\sigma }^{2}$.

## Results

### Data description

The data descriptions of dengue cases for each study district are given in Table 2.

**Table 2:** Summary measures of dengue cases across study regions.

Region	Hulu Selangor	Petaling	Klang	Sepang	Hulu Langat	Gombak	Kuala Selangor
**Dengue cases**							
Average	7.48	98.46	41.25	6.63	77.11	44.95	5.87
[Range]	[0, 47]	[14, 676]	[10, 150]	[0, 61]	[17, 405]	[4, 174]	[0, 26]

Prior to model building and selection, the value of dengue cases of each study region is normalized into the same range (0–1): Letting $A$ represent a generic notation for a variable which represents number of cases, normalization entails the formula given in Eq. (5).

5 $${A}_{N}=\frac{A-{A}_{min}}{{A}_{max}-{A}_{min}}$$

### Single dengue prediction models

#### Phase I: ARIMA model development

**Figure 3:**
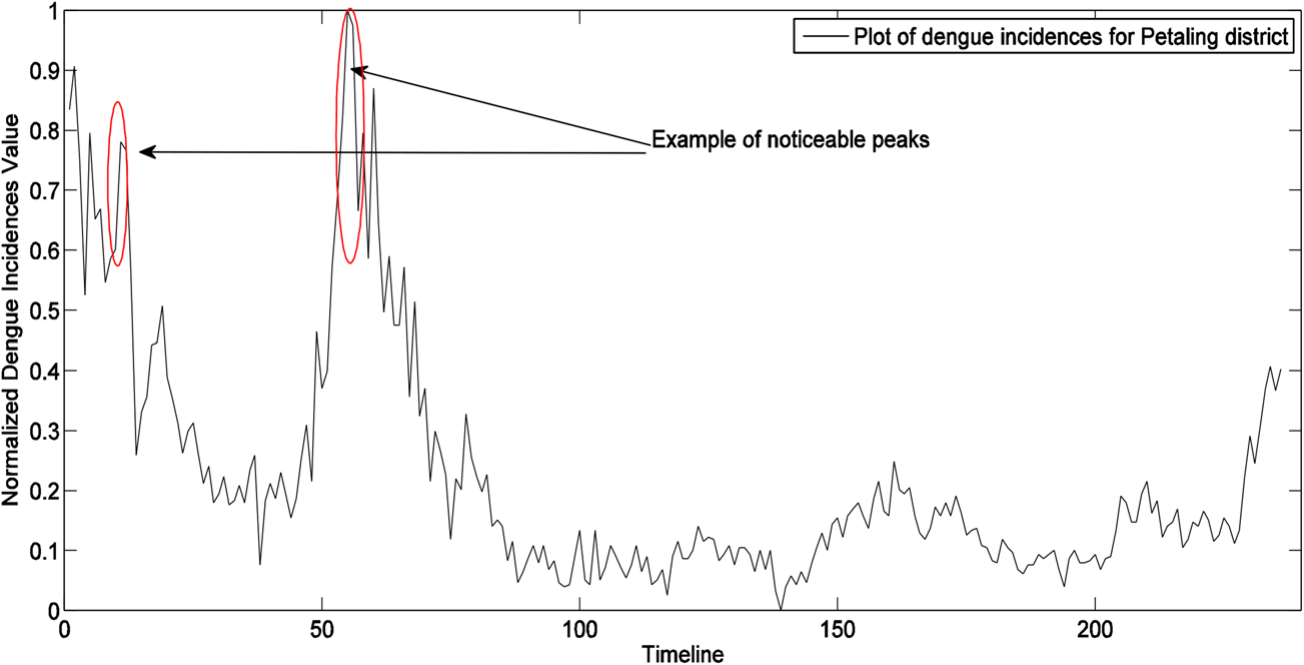
Plot dengue cases for Petaling district.

**Figure 4:**
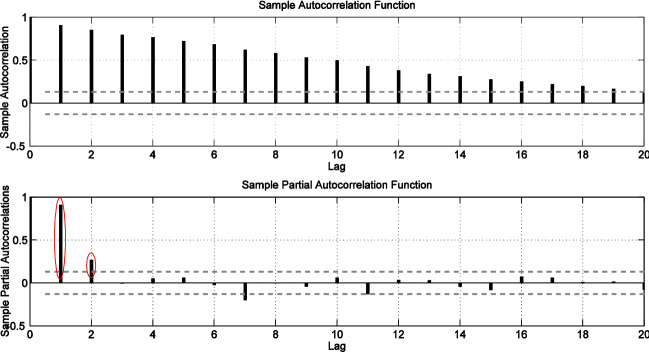
Plot of ACF and PACF for Petaling district.

**Figure 5:**
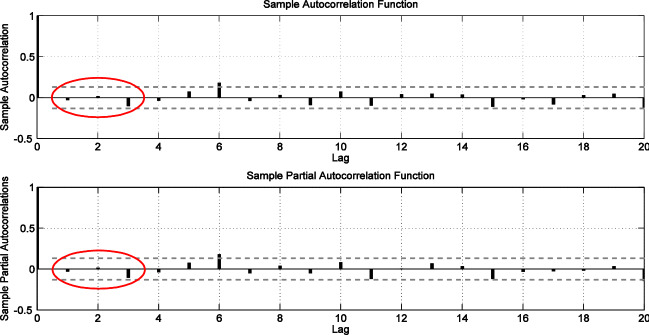
Residual Plot of ACF and PACF for Petaling district ARIMA (2,1,0).

The development of the dengue prediction model is illustrated for Petaling district in Figs. 3, 4 and 5. Figure 3 shows the weekly dengue case trajectory for Petaling district during years of 2009–2013 which shows clearly the cases are not stationary as they have many noticeable peaks. First order differencing (*d* = 1) was carried out and visible lag orders are identified using the ACF and PACF plots. The PACF plot in Fig. 4 shows significant peaks at *p* = 1 and *p* = 2, hence, ARIMA (2,1,0) model is selected as a viable option. Figure 5 shows the ACF and PACF plots which depicts residuals from the fitted model have no significant peaks outside the 95% confidence intervals for the first three lags, indicating that the residuals are possibly white noise. Hence, ARIMA (2,1,0) model structure is set for dengue cases of Petaling district (taking the case for a district).

### Ensemble ARIMA model development

Single dengue models are enlarged by including its neighbouring districts’ (as in Table 1), forming the ensemble dengue models. Single models are built using dengue cases time series from its own district, whereas ensemble models are built based on combining own district, and neighbouring districts’ dengue models. The ensemble ARIMA model, by taking the example of Petaling district is described as in Eq. (6).

6 $$\begin{aligned} en - ARIMA \left( {2,1,0,X} \right)_{P} & = ARIMA \left( {2,1,0} \right)_{P} + \left( X \right) \\ en - ARIMA \left( {2,1,0,X} \right)_{P} & = ARIMA \left( {2,1,0} \right)_{P} + \left( {\alpha \hat{Y}_{HL} + \beta \hat{Y}_{G} + \gamma \hat{Y}_{KS} + \delta \hat{Y}_{K} } \right) \\ \end{aligned}$$

where $ARIMA {\left(\mathrm{2,1},0\right)}_{P}$ is the ARIMA (2,1,0) model using observations from the dengue prediction model of Petaling district, the exogenous (X) part is built using observations from the four neighbouring regions’ ARIMA dengue prediction models of Hulu Langat, Gombak, Kuala Selangor and Klang: $\widehat{HL}, \widehat{G},$ $\widehat{KS}$ and $\widehat{K}$, respectively. These observations are obtained from the respective single models, which are the ARIMA (1,1,2) * model for Hulu Langat district, ARIMA (2,1,0) model for both Gombak and Klang districts and finally ARIMA (3,1,0) for Kuala Selangor district. Parameters $\alpha , \beta ,$ $\gamma$ and $\delta$ are the coefficients of the respective dengue prediction models.

### Comparisons between single and ensemble ARIMA dengue prediction models

**Figure 6:**
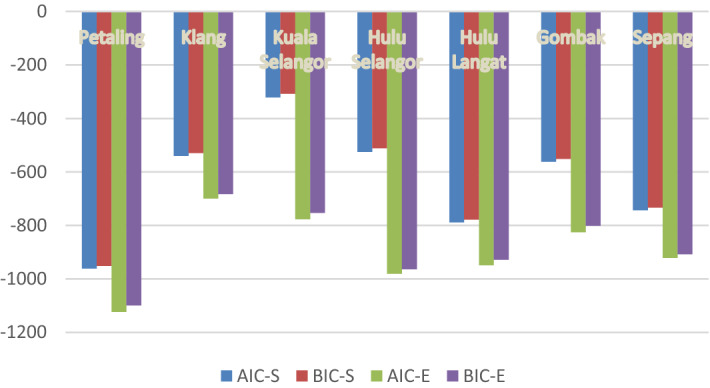
Comparison between ARIMA and ensemble ARIMA models.

Figure 6 shows the results obtained for single (AIC-S and BIC-S) and ensemble ARIMA model (AIC-E and AIC-E) comparisons. The results obtained indicate that ensemble models have lower AIC and BIC values in all of the seven districts considered (refer to Table 3). This explains that across all seven study districts, ensemble models have better fit and predicts dengue cases better compared to single dengue models.

**Table 3:** A Comparison between single and ensemble dengue climate prediction models.

No	Study area	Dengue prediction models			
		Single		Ensemble	
		Best model structure	AIC, (BIC)	Best Model Structure	AIC, (BIC)
1	Petaling	ARIMA (2,1,0)	− 961.5 (− 951.1)	ARIMA (2,1,0,$X$)	− 1,123.2 (− 1,099.2)
2	Klang	ARIMA (2,1,0)	− 540.3 (− 529.9)	ARIMA (2,1,0,$X$)	− 699.9 (− 682.7)
3	Kuala Selangor	ARIMA (3,1,0)	− 321.3 (− 307.5)	ARIMA (3,1,0,$X$)	− 776.9 (− 752.8)
4	Hulu Selangor	ARIMA (3,1,0)	− 525.1 (− 511.3)	ARIMA (3,1,0,$X$)	− 981.0 (− 963.8)
5	Hulu Langat	ARIMA (1,1,2)*	− 788.6 (− 778.2)	ARIMAX (1,1,2,${X}_{4}$)*	− 987.3 (− 966.8)
6	Gombak	ARIMA (2,1,0)	− 561.8 (− 551.4)	ARIMA (2,1,0,$X$)	− 825.1 (− 801.0)
7	Sepang	ARIMA (2,1,0)	− 743.5 (− 733.1)	ARIMA (2,1,0,$X$)	− 921.3 (− 907.5)

## Discussion

This finding explains that the pattern of dengue cases at neighbouring districts influences and suggests that human movements and contact between neighbouring districts is a factor in dengue transmission and the spread of the disease. Human mobility is neighbouring areas  allows for the possibility of an infected person to transmit dengue virus to the surrounding areas^26^. Previous studies have shown that urban areas with high population densities and with increased movement of infected individuals contributes to increase spread of the disease^27–29^. Sources for human mobility can be obtained from the geo-location information in mobile phone data^30–32^ and from data on the use of public transportation^33,34^, air travel patterns and data on human mobility^35^. In all these studies, authors managed to conclude that inclusion of human mobility data enhanced the dengue prediction models which is shown as ensemble of neighbouring regions in this study.

## Conclusion

Firstly, modelling dengue cases using ARIMA models in this study, is considered crucial and appropriate since these models can incorporate feedback information. Feedback information is necessary for modelling infectious disease trajectories, such as dengue, whose current values are strongly correlated to past values. In order to fit ARIMA models to dengue trajectories which are typically non-stationary, one has to first apply differencing in order to convert these times series into stationary ones^36,37^.

Based on the single-ensemble model comparisons, this study concludes that dengue cases are subject to spatial effects, that is, patterns of dengue cases in neighboring areas do influence dengue case pattern of the study area.

Among the limitation of this study is that besides spatial effects, there are many other factors that may influence dengue cases. These include climate, new serotypes, herd immunity and strain-cross immunity, impact of vector control programs that have been carried out and finally mosquito vector densities^38,39^. A better fit model can be obtained by including all these factors.

Finally, dengue prediction models in this study are only built for seven districts in Selangor as incorporating larger area may not yield an optimal model given that the dengue prediction models are localized, where they only describe their own respective district’s dengue case patterns. Future work shall investigate other districts in other states in Malaysia, especially Wilayah Persekutuan, Kuala Lumpur and Johor which have reported high number of dengue cases^39^.
